# Pannexin3 inhibits TNF‐α‐induced inflammatory response by suppressing NF‐κB signalling pathway in human dental pulp cells

**DOI:** 10.1111/jcmm.12988

**Published:** 2016-09-29

**Authors:** Fangfang Song, Hualing Sun, Yake Wang, Hongye Yang, Liyuan Huang, Dongjie Fu, Jing Gan, Cui Huang

**Affiliations:** ^1^ The State Key Laboratory Breeding Base of Basic Science of Stomatology (Hubei‐MOST) & Key Laboratory of Oral Biomedicine Ministry of Education (KLOBM)School & Hospital of StomatologyWuhan UniversityWuhanHubeiChina; ^2^ Department of StomatologyRenmin Hospital of Wuhan UniversityWuhanHubeiChina

**Keywords:** TNF‐α, pulpitis, human dental pulp cells, Pannexin3, proteasome, NF‐κB

## Abstract

Human dental pulp cells (HDPCs) play a crucial role in dental pulp inflammation. Pannexin 3 (Panx3), a member of Panxs (Pannexins), has been recently found to be involved in inflammation. However, the mechanism of Panx3 in human dental pulp inflammation remains unclear. In this study, the role of Panx3 in inflammatory response was firstly explored, and its potential mechanism was proposed. Immunohistochemical staining showed that Panx3 levels were diminished in inflamed human and rat dental pulp tissues. *In vitro*, Panx3 expression was significantly down‐regulated in HDPCs following a TNF‐α challenge in a concentration‐dependent way, which reached the lowest level at 10 ng/ml of TNF‐α. Such decrease could be reversed by MG132, a proteasome inhibitor. Unlike MG132, BAY 11‐7082, a NF‐κB inhibitor, even reinforced the inhibitory effect of TNF‐α. Quantitative real‐time PCR (qRT‐PCR) and enzyme‐linked immunosorbent assay (ELISA) were used to investigate the role of Panx3 in inflammatory response of HDPCs. TNF‐α‐induced pro‐inflammatory cytokines, interleukin (IL)‐1β and IL‐6, were significantly lessened when Panx3 was overexpressed in HDPCs. Conversely, Panx3 knockdown exacerbated the expression of pro‐inflammatory cytokines. Moreover, Western blot, dual‐luciferase reporter assay, immunofluorescence staining, qRT‐PCR and ELISA results showed that Panx3 participated in dental pulp inflammation in a NF‐κB‐dependent manner. These findings suggested that Panx3 has a defensive role in dental pulp inflammation, serving as a potential target to be exploited for the intervention of human dental pulp inflammation.

## Introduction

Dental pulp inflammation is a widespread public problem and is commonly a sequel to caries or trauma 1. In clinical setting, such inflammation always causes severe pain, of which level has been anecdotally considered as “the highest level possible” 2, 3, and if kept uncontrolled, it may finally even result in fatal systemic inflammatory disorders 3, 4. Considering the emergent influence of pulpitis, understanding the pathological progression of pulpitis is of great interest and is an indispensable premise to its effective management. However, the mainstream studies have primarily emphasized on the roles of immune cells 5, 6, such as dendritic cells, macrophages and lymphocytes. However, this is far from exhaustive.

Actually, human dental pulp cells (HDPCs), the major cell types in dental pulp, play a broad variety of functions in host defence and regeneration 7, 8, 9. Upon stimulation with pro‐inflammatory mediators, including TNF‐α, HDPCs secrete significant amounts of cytokines locally for attracting additional immune cells as well as initiating and modulating inflammatory responses 8, 10. TNF‐α, which has been documented as an objective marker of early inflammation, plays important roles in the inflammatory response 10, 11, 12, 13.

Pannexins (Panxs) is a family of transmembrane channels that assemble to form hemichannels. Hemichannels could direct cell‐matrix communication through the exchange of small molecules, including ATP, NAD+, Ca^2+^ and glutamate, respectively 14. This behaviour might enable the heterogeneous dental pulp tissues to act as an integrated system. The Panxs family consists of three types: Pannexin1 (Panx1), Pannexin2 (Panx2) and Pannexin3 (Panx3). Panxs is reported to play important roles in co‐ordinating tissue homeostasis and regulating the pathogenesis of various inflammation statuses 15. As a signal conductor and inflammatory mediator, Panx3 could release nucleotides, which could regulate the crosstalk between muscle cells and immune cells, upon metabolic inflammation stimulation 16. In addition to the hemichannel function, Panx3 participates in several intracellular signalling pathways, and regulates gene expression and cell activity 17, 18. In addition, our previous study has demonstrated that Panx3 was expressed in human dental pulp tissues and human odontoblastic‐like cells 19. These inspire us to hypothesize that single Panx3 plays critical roles during dental pulp inflammation. However, to the best of our knowledge, no report is available until now.

Herein, we report that Panx3 was down‐regulated in inflamed human and rat dental pulp tissues. Panx3 regulated the expression of IL‐1β and IL‐6 in response to TNF‐α stimulation by mediating the NF‐κB signalling pathway. The immunosuppressive role of Panx3 may be used for therapeutic prevention of dental pulp inflammation.

## Material and methods

### Ethical statements

All experiments were approved by the Institutional Ethical Board of Wuhan University and were done followed by the guide lines of the National Institute of Health (NIH).

### Collection of human dental pulp tissues

Human dental pulp samples were collected from 12 patients (mixed gender) aged from 18 to 32 years old, including of 6 cases of healthy third molar teeth and 6 cases of pulpitis teeth, with informed consent from the School and Hospital of Stomatology, Wuhan University. The cases of dental pulpitis were diagnosed by endodontic specialists on the basis of established criterion before the root canal therapy 20, referring to those with a history of spontaneous, intense and prolonged pain without periodontal disease 11. Each group was used for H&E and immunohistochemical staining.

### Induction of pulpitis

Eight 8‐week‐old male Wistar rats were purchased from the Hubei Research Center of Laboratory Animals and kept in a temperature‐ and light‐controlled animal room in all experiments. We divided the rats into two groups randomly: experimental groups (*n* = 4) and normal (*n* = 4). The rats were anesthetized with 10% chloral hydrate (0.33 ml/100 g bodyweight) *via* intraperitoneal injection. The maxillary first and second molars were drilled open on the occlusal face by a low‐speed dental drill without cooling 8. The normal group only performed the same process of anaesthesia without any other operation. Rats were killed at 24 hrs post‐operatively, and the upper jaws were extracted.

### H&E staining and immunohistochemical staining of dental pulp

Healthy human and rat teeth and inflamed rat teeth were promptly fixed in 4% buffered para‐formaldehyde for 24 hrs at room temperature, decalcified using 10% EDTA for 3 months, and then dehydrated and embedded in paraffin. The inflamed human dental pulp samples were pulled out of the cavity and fixed in the 4% para‐formaldehyde for 24 hrs immediately. Serial sections 5 μm thick were cut, mounted on silane‐coated slides (Thermo, Waltham, MA, USA), then deparaffinized and rehydrated. H&E and immunohistochemical staining were separately performed on consecutive tissue sections. Immunohistochemical staining was performed following the manufacturer's recommended protocols (Zhong‐Shan Biotech, Beijing, China). The sections were treated with gastric enzyme for antigen retrieval and subsequently incubated in a bathing solution of 3% hydrogen peroxide for endogenous peroxidases block. The sections were blocked with 5% goat serum and incubated with primary rabbit polyclonal antibody against Panx3 (1:50, Santa Cruz, Texas, CA, USA) and mouse monoclonal antibody against TNF‐α (1:50, PMK Bioechnology co., LTD, Wuhan, China) overnight at 4°C. Slides were then treated with corresponding secondary biotinylated goat anti‐rabbit antibody and secondary biotinylated goat antimouse antibody for 15 min. at 37°C. Diaminobenzidine‐HCl (DAB) was used as a substrate for visualization. Finally, the nuclei were counterstained with haematoxylin.

### Cultivation and treatment of HDPCs

HDPCs derived from human healthy premolar or third molar teeth of 18‐ to 24‐year‐old patients (mixed gender) with informed consent were used in this study. The dental pulps were pulled out of the cavity, minced into small pieces and digested with collagenase/dispase enzyme (Roche, Basel, Switzerland). Cells were grown in α‐modified essential medium (HyClone, South Logan, UT, USA) supplemented with 10% fatal bovine serum (FBS, Gibco, Grand Island, NY, USA), 100 U/ml penicillin and 100 U/ml streptomycin, under humidified atmosphere with 5% CO_2_ at 37°C. HDPCs at passage 3–5 were used in the following experiments. In the particular experiments, cells were treated with recombinant human TNF‐α (Peprotech, Rocky Hill, NJ, USA) or pre‐treated with BAY 11‐7082 (a NF‐κB inhibitor, 2 μM, Beyotime Biotechnology, Shanghai, China) or MG132 (a proteasome inhibitor, 1 μM, Selleckchem, Houston, TX, USA) before TNF‐α stimulation for the given time.

### Cell immunofluorescence

HDPCs were plated onto coverslips at a density of 2 × 10^4^ cells/cm^2^. After 24 hrs, the cells were treated with TNF‐α for respective timescales. Briefly, the cells were fixed in 4% paraformaldehyde for 10 min. at room temperature, washed in PBS, permeabilized with 0.1% triton for 5 min. and blocked with 4% BSA for 30 min. at 37°C. Subsequently, the slides were incubated with primary rabbit polyclonal antibody against Panx3 (1:25; Invitrogen, Carlsbad, CA, USA) and rabbit monoclonal antibody against NF‐κB‐p65 (1:100; Cell Signaling Technology, Danvers, MA, USA) at 4°C in a moist chamber overnight. After three washes, the slides were incubated with DyLight 549‐conjugated goat anti‐rabbit antibody (1:200; Abbkine, Redlands, CA, USA) for 60 min. at 37°C. After three washes, nuclei were then stained with 4, 6‐diamidino‐2‐phenylindole (DAPI). For control, the primary antibody was omitted.

### Cloning of human Panx3

Total RNA was extracted from HDPCs using Trizol (Invitrogen, USA), reverse transcribed using PrimeScript^™^ RT Reagent Kit with gDNA Eraser (Takara, Japan). Full‐length cDNA for human Panx3 was obtained by PCR using PrimeSTAR Max DNA polymerase (Takara, Japan) and the followed primers: forward: 5′‐CCGGAATTCatgtcacttgcacacaca‐3′ and reverse: 5′‐CGCGGATCCaagctttcttgctccat‐3′ containing extra EcoRI and BamHI restriction sites. The PCR product was purified by gel extraction kit (CWBIO, Beijing, China) and subsequently cloned into an empty pLVX‐IRES‐ZsGreen1 (Clontech, Mountain View, CA, USA) plasmid. The final construct was sequenced for verification, and named PLVX‐Panx3 plasmid.

### Lentiviral package and transduction

Panx3 was overexpressed using above‐mentioned Plvx‐Panx3 plasmid and suppressed using shRNA targeting the human Panx3 gene (Genechem, GUAGCAAUAUACACCAUAUTT). pLVX‐IRES‐ZsGreen1 and nonsense shRNA were used as control. 293E cells were plated onto 10 cm dish at a density of 4 × 10^6^ cells/plate. The three‐plasmid systems, including pMD2.G and psPAX2, were co‐transfected according to the manufacturer's instruction of TurboFect (Thermo, USA). After 48 hrs, the lentiviral particles were collected, centrifuged and filtered with a 0.45 μm filter. For infection, HDPCs were incubated with the lentivirals for 12 hrs containing 5 μg/ml polybrene. The cells were then named HDPC/Panx3, HDPC/Plvx, HDPC/shRNA and HDPC/Ctrl. After 48 hrs, photographs of the GFP‐positive cells were obtained using a fluorescent microscope, and the expression of Panx3 was quantified by qRT‐PCR and Western blot.

### NF‐κB luciferase assay

HDPCs were plated into 24‐well plates at a density of 3 × 10^4^ cells/well. Lipofectamine 2000 (Thermo, USA) was used for co‐transfection with 0.5 μg reporter plasmids pNF‐κB‐luc and 0.05 μg internal control plasmids pRL‐TK (Promega, Madison, WI, USA). After 6 hrs, the cells were washed with PBS and changed with fresh mediums containing 10 ng/ml TNF‐α for another 24 hrs. Then the cells were lysed with Passive Lysis Buffer (Promega, USA) for 15 min. on the shaker. The firefly and Renilla luciferase activities were assessed according to the manufactures’ instruction using GloMax^®^ 20/20 Luminometer (Promega, USA). The relative NF‐κB transcriptional activity (relative light units of firefly luciferase/relative light units of Renilla luciferase, fRLU/rRLU) was calculated.

### qRT‐PCR

Total RNA was extracted from HDPCs using Trizol (Invitrogen, USA). One microgram RNA was used to transcribe into cDNA which acted as the template for the qRT‐PCR using PrimeScript^™^ RT Reagent Kit with gDNA Eraser (Takara, Japan). qRT‐PCR reaction was performed with SYBR^®^ Premix Ex Taq II (Tli RNaseH Plus) (Takara, Tokyo, Japan) on an ABI 7900 fast system (Applied Biosystems, Foster City, CA, USA). The primers were listed in Table 1. The values were calculated applying the 2^−∆∆Ct^ formula. GAPDH was used as the internal control. The experiments were repeated three times.

**Table 1:** Primer sequences for qRT‐PCR

Gene	Forward primer (5′–3′)	Reverse primer (5′–3′)
Panx3	ATCATCAGCGAACTGGACAAAT	AAGTATCGTTCTTTCCGAGCCT
TNF‐α	GTCACTCATTGCTGAGCCTCT	AGCTTCTTCCCACCCACAAG
IL‐1β	TCAATATTAGAGTCTCAACCCCCA	TTCTCTTTCGTTCCCGGTGG
IL‐6	CATCACCATCTTCCAGGAG	AGGCTGTTGTCATACTTCTC
GAPDH	TGAACGCTGCTTACATGCCA	AGGCTGTTGTCATACTTCTC

Panx3, Pannexin 3.

### Western blot

HDPCs were lysed with RIPA buffer containing PMSF (Roche, Germany) and phosphatase inhibitors (Roche, Germany), and then centrifuged at 12,000 × g for 15 min. at 4°C. BCA assay kit (Biosharp, Hefei, China) was used to determine protein concentration and then equal total protein was denatured by heating at 95°C for 5 min. Total 30 μg protein was electrophoresed on 10% SDS‐polyacrylamide gel and transferred onto PVDF membrane (Millipore, Billerica, MA, USA). The membranes were blocked with 5% non‐fat milk for 60 min. at room temperature, and then incubated with primary rabbit polyclonal antibody against human Panx3 (1:300; Invitrogen), primary rabbit polyclonal antibody against human phosphorylated‐p65^S536^ (1:1000; Cell Signaling Technology), and mouse monoclonal antibody against IκBα (1:1000; Cell Signaling Technology) on shaker overnight at 4°C. After three washes with TBST, the membranes were incubated with HRP‐conjugated secondary antibody at room temperature for 1 hr. The Protein bands were visualized using ECL reagent, detected with X‐ray film and quantified by densitometry using Image J (National Institutes of Health, Bethesda, Maryland, USA). GAPDH was used as the internal control. Western blot experiments were repeated at least three times.

### ELISA

HDPCs were plated into 24‐well plates at a density of 8 × 10^4^ cells/well. The supernatants of HDPCs treated with and without TNF‐α for 24 hrs were collected. Human IL‐1β and IL‐6 concentration in the supernatants were quantitatively measured using a commercial ELISA kit (Boster, Wuhan, China) following the manufacturer's instructions. The HDPCs were lysed with 2% Triton‐X 100, and the protein concentrations were determined using a BCA assay kit. The IL‐1β and IL‐6 concentrations were normalized to 1 ug protein. ELISA experiments were repeated at least three times.

### Statistical analysis

All the quantitative data were presented as mean ± SEM. Comparisons were analysed by Student's *t*‐test for two groups and anova for multi‐group using SPSS version 17.0. *P* < 0.05 was considered significantly different.

## Results

### Panx3 is decreased in human and rat dental pulpitis specimens

**Figure 1:**
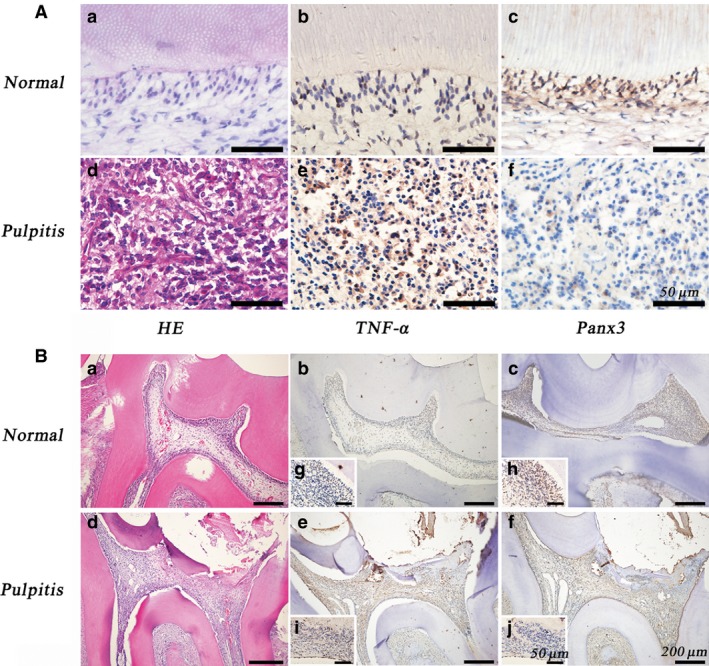
The expression of Panx3 was decreased in inflamed human (**A**) and rat dental pulpitis tissue (B). The dental pulp tissue sections were stained with HE, anti‐Panx3 antibody or anti‐TNF‐α antibody. HE‐stained sections of normal and inflamed dental pulp tissue verified the progression of inflammation. Immunohistochemistry performed on dental pulp tissues showed the presence of Panx3 in normal tissue, but decreased in the inflamed pulp tissue. In contrast to Panx3 expression, TNF‐α was increased in inflamed dental pulp tissue. Cell nuclei were visualized with haematoxylin. Scale bars are stamped in the images, respectively.

The differences in Panx3 expression between healthy and inflamed pulp tissues built the prerequisite for all subsequent experiments. Dental pulp tissues were isolated from healthy control individuals and dental pulpitis patients, and their expressions of Panx3 and TNF‐α were detected by immunohistochemistry staining (Fig. 1A). H&E staining revealed increased immune cells in pulpitis tissues (Fig. 1Aa, d). Immunohistochemistry staining showed that Panx3 clearly expressed in the odontoblast layer of normal dental pulp tissue (Fig. 1Ac). Notably, Panx3 was seldom present at the inflammatory sites (Fig. 1Af). Accompanying the progression of inflammation, TNF‐α expression was elevated in local inflammatory region (Fig. 1Ab, e).

We next performed immunohistochemistry to examine the expression of Panx3 in rat normal and inflamed dental pulp tissues (Fig. 1B). H&E staining was performed to identify the histopathological changes in dental pulp, revealing that increased immune cells were present in experimental groups 24 hrs post‐operatively (Fig. 1Ba, d). Previously, it was reported that pulpal exposure for 24 hrs could result in dental pulp inflammation and has been used to establish the model of dental pulpitis in several studies 2, 8. Similar to above‐mentioned results, Panx3 was located in the odontoblast layer of rat normal dental pulp tissue, and was decreased at the inflammatory sites (Fig. 1Bc, f, h, j). TNF‐α expression profile was similar to those in human tissues (Fig. 1Bb, e, g, i).

### TNF‐α concentration dependency of Panx3 expression in HDPCs

**Figure 2:**
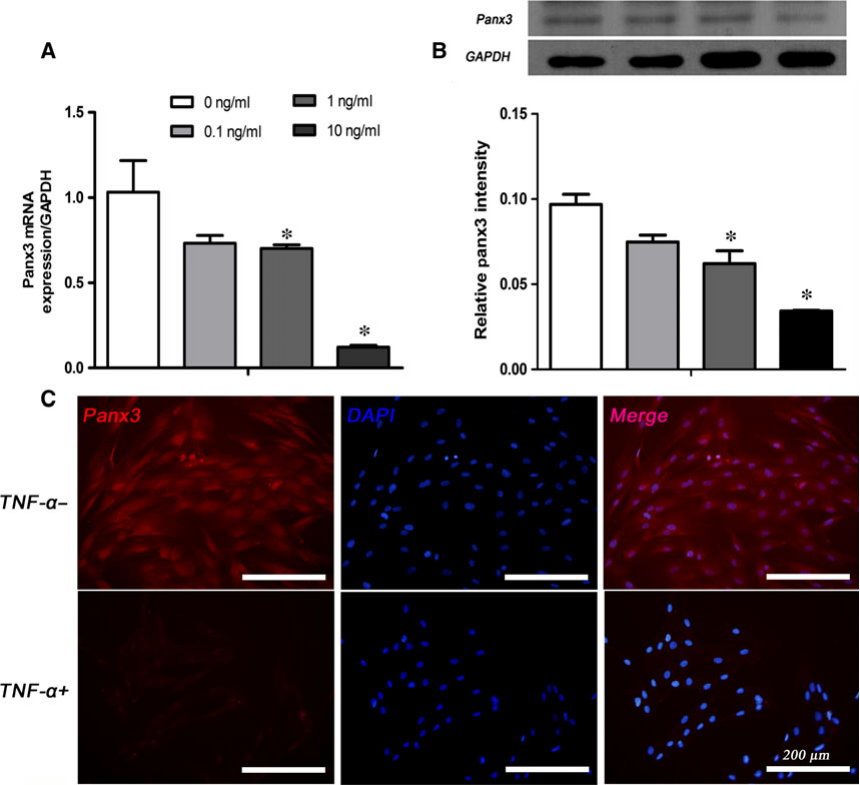
TNF‐α repressed Panx3 expression at the mRNA and protein levels in a concentration‐dependent manner in HDPCs. The mRNA and protein expression levels of Panx3 in HDPCs stimulated with TNF‐α (0–10 ng/ml) for 24 hrs were detected by qRT‐PCR (**A**) and Western blot (**B**). GAPDH served as an internal control. (**C**) Immunofluorescence of Panx3 in HDPCs with or without TNF‐α (10 ng/ml) treatment for 24 hrs. Scale bar = 200 μm. Data were expressed as mean ± SEM. *P < 0.05 compared to the group treated without TNF‐α.

Considering the question was whether pro‐inflammatory cytokines impacted Panx3 expression levels, HDPCs were incubated with various concentration of TNF‐α (0, 0.1, 1, 10 ng/ml) for 24 hrs, then their Panx3 levels were analysed by qRT‐PCR, Western blot and immunofluorescence (Fig. 2A–C). After treatment with TNF‐α, a dose‐dependent inhibitory effect of TNF‐α on Panx3 was observed, mRNA expression being reduced by 25%, 30% and 70% at concentrations of 0.1, 1 and 10 ng/ml of TNF‐α comparable to the baseline, respectively (Fig. 2A). In parallel, Western blot results also showed similar trends in Panx3 protein level, which reached a minimum at 10 ng/ml of TNF‐α (Fig. 2B). Therefore, the specific concentration of TNF‐α (10 ng/ml) was chosen for all subsequent experiments. Corresponding results could also be observed in immunofluorescence assay, indicating that the level of Panx3 was evidently lower after treatment with TNF‐α for 24 hrs. Immunofluorescence results showed that Panx3 was expressed in the cell membrane and cytoplasm (Fig. 2C), which was consistent with previous study that Panx3 is expressed in plasma membrane and diffusely in the cytosol acting as and ER Ca^2+^ channel 19, 21.

### Proteasome inhibitor not NF‐κB inhibitor partially reversed the effects of TNF‐α on Panx3 expression

**Figure 3:**
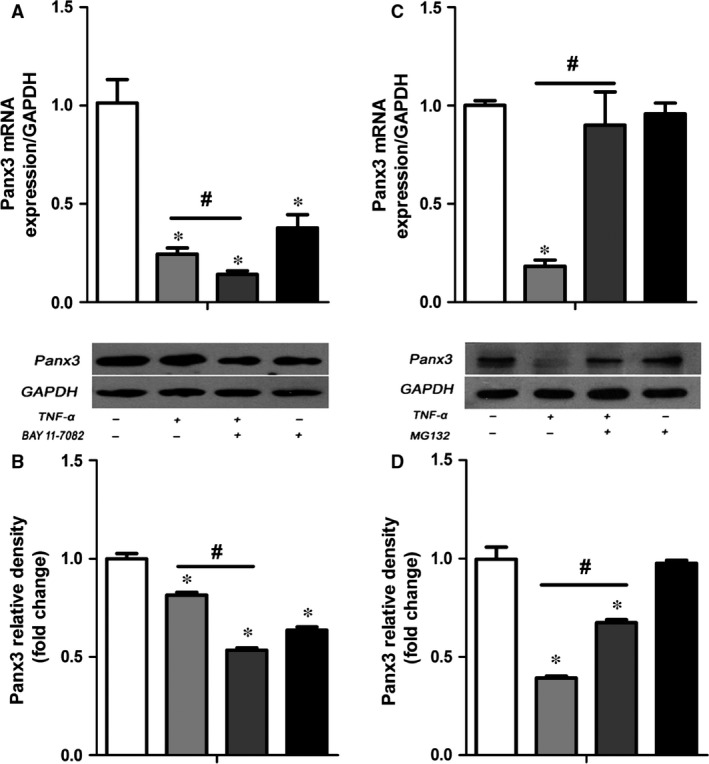
MG132 not BAY 11‐7082 rescued the TNF‐α‐induced down‐regulation of Panx3. Cells were pre‐treated with 2 μM BAY 11‐7082, 1 μM MG132 or DMSO for 30 min. before TNF‐α treatment. qRT‐PCR (**A, C**) and Western blot analysis (**B, D**) were then performed to determine the Panx3 expression. *P < 0.05 compared to the group without stimulation. ^#^ *P* < 0.05 compared to the group treated with TNF‐α alone.

To gain insight into the mechanisms underlying TNF‐α–down‐regulated Panx3 expression, cells were pre‐treated with specific inhibitor BAY 11‐7082 (2 μM) and MG132 (1 μM) for 30 min. before TNF‐α stimulation. Notably, qRT‐PCR and Western blot results showed that instead of reversing the repressed expression of Panx3, BAY 11‐7082 even exacerbated the inhibitory effect (Fig. 3A, B). Although not completely abrogated, the inhibitory effect was evidently rescued by MG132 (Fig. 3C, D). DMSO was used as vehicle. Thus, it is elucidated that TNF‐α inhibits Panx3 expression *via* proteasome pathway, while NF‐κB inactivation reduces the expression of Panx3.

### Panx3 overexpression or knockdown in HDPCs

**Figure 4:**
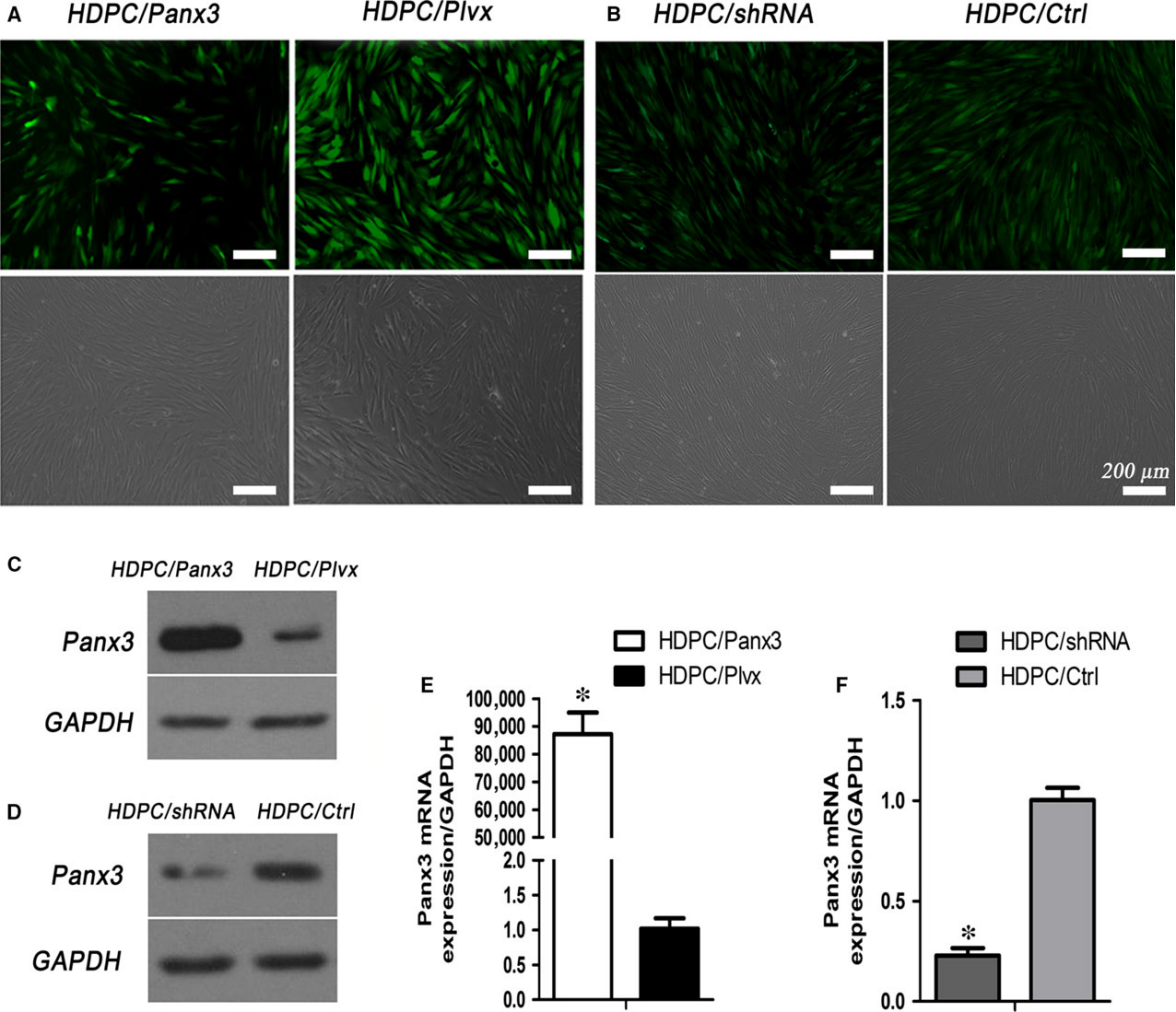
Lentiviral‐mediated expression of Panx3 in HDPCs. (**A, B**) Cell images of HDPCs/Panx3, HDPC/Plvx, HDPC/shRNA and HCPD/Ctrl groups were photographed in normal light (lower panels) and under a fluorescence microscope (upper panels). Protein and mRNA expression of Panx3 were determined by Western blot (**C, D**) and qRT‐PCR (**E, F**) analysis. *P < 0.05 HDPC/Panx3 *versus* HDPC/Plvx, and HDPC/shRNA *versus* HDPC/Ctrl. Scale bar: 200 μm.

Transfection of HDPCs with Plvx‐Panx3 lentivirus effectively resulted in overexpression of Panx3, and the ratio of GFP‐expressed cells/total cells was almost 70% (Fig. 4A). The overexpression efficiency was confirmed by Western blot and qRT‐PCR (Fig. 4C, E). Panx3 mRNA was increased by over 80,000‐fold (Fig. 4E), followed by elevated content of Panx3 protein (Fig. 4C). A lentiviral system harbouring shRNA was used to knockdown Panx3, and the ratio of GFP‐expressed cells/total cells was almost 80% (Fig. 4B). The Western blot and qRT‐PCR results showed that the protein level of Panx3 was significantly decreased and the mRNA expression was declined by over 75% (Fig. 4D, F). Cells infected with empty pLVX‐IRES‐ZsGreen1 plasmid and nonsense sequence shRNA were used as control.

### Functional impact of Panx3 overexpression and knock down

**Figure 5:**
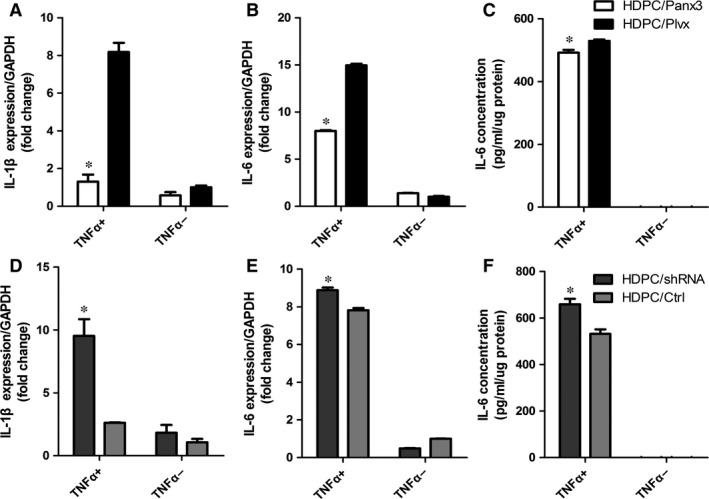
Panx3 regulates TNF‐α‐induced inflammatory cytokine expression in HDPCs. qRT‐PCR (**A, B**) and ELISA (**C**) analysis of IL‐1β and IL‐6 expression in HDPC/Panx3 and HDPC/Plvx cells upon TNF‐α stimulation for 24 hrs. qRT‐PCR (**D, E**) and ELISA (**F**) analysis of IL‐1β and IL‐6 expression in HDPC/shRNA and HDPC/Ctrl cells upon TNF‐α stimulation for 24 hrs. *P < 0.05 HDPC/Panx3 *versus* HDPC/Plvx, and HDPC/shRNA *versus* HDPC/Ctrl.

Having established the transfection efficiency, the effect of Panx3 expression on TNF‐α‐induced inflammatory response was examined. HDPC/Panx3, HDPC/Plvx, HDPC/shRNA and HDPC/Ctrl cells were, respectively, treated with or without TNF‐α (10 ng/ml) for 24 hrs, and their levels of IL‐1β and IL‐6 were then measured. Overexpressed Panx3 resulted in a robust suppression of IL‐1β and IL‐6 expression (Fig. 5A–C). Confirming these findings, TNF‐α stimulation in the Panx3 knockdown HDPCs was followed by an evident increase in IL‐1β and IL‐6 (Fig. 5D–F). IL‐1β protein in the supernatants was lower than the detection range of the ELISA. Effects regarding the alteration of IL‐1β and IL‐6 after knockdown of Panx3 were consistent with results from the overexpression experiments, emphasizing the conclusion that Panx3 mediates pro‐inflammatory capacity of HDPCs.

### Panx3 negatively regulates NF‐κB signalling pathway

**Figure 6:**
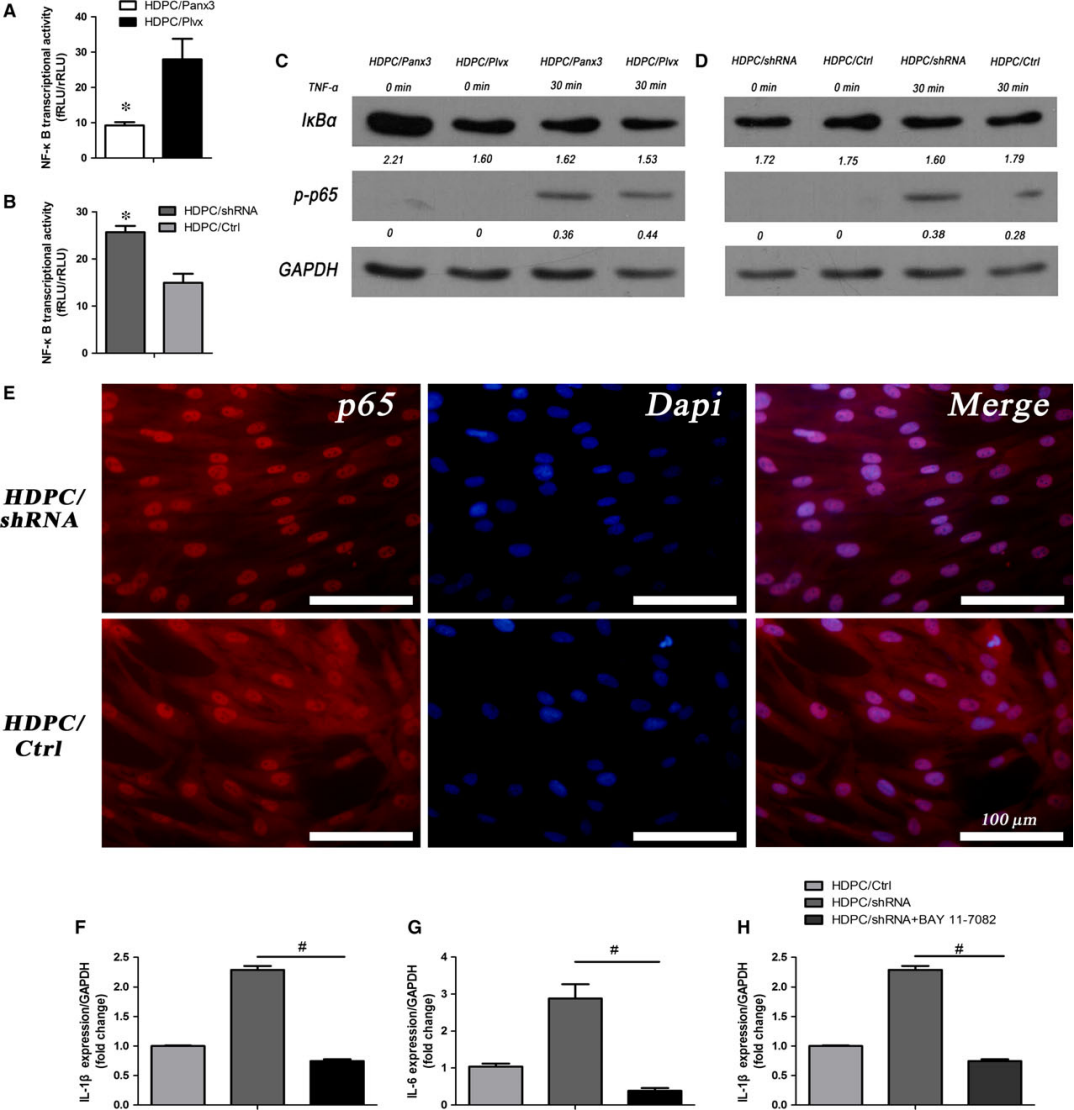
Panx3 regulates NF‐κB signalling pathway. (**A, B**) NF‐κB transcriptional activities in HDPC/Panx3, HDPC/Plvx, HDPC/shRNA and HDPC/Ctrl groups were measured using dual‐luciferase reporter system. *P < 0.05 HDPC/Panx3 *versus* HDPC/Plvx, HDPC/shRNA *versus* HDPC/Ctrl. (**C, D**) Phosphorylated‐p65 and IκBα protein expression were verified by Western blot in HDPC/Panx3, HDPC/Plvx, HDPC/shRNA, HDPC/Ctrl groups with or without TNF‐α stimulation for 30 min. GAPDH was used as the internal control. (**E**) Elevated nucleus translocation of NF‐κB‐p65 in TNF‐α‐treated HDPCs from the Panx3 silenced group (HDPC/shRNA) compared with HDPC/Ctrl, detected by immunofluorescence. *P < 0.05 HDPC/Panx3 *versus* HDPC/Plvx, and HDPC/shRNA *versus* HDPC/Ctrl. Scale bar = 100 μm. (**F–H**) Inflammatory cytokines expression were quantified by qRT‐PCR and ELISA in HDPC/shRNA, HDPC/Ctrl and HDPC/shRNA‐BAY 11‐7082 groups. ^#^ *P* < 0.05 HDPC/shRNA‐ BAY 11‐7082 *versus* HDPC/shRNA.

Addressing the potential mechanism involved in TNF‐α‐induced pro‐inflammatory cytokines production, alterations of the NF‐κB pathway were further examined. The dual‐luciferase reporter assay results showed that Panx3 overexpression could decrease the luciferase activity of the NF‐κB activation for almost 60% compared with HDPC/Plvx, whereas those of Panx3 knockdown increased to 1.7‐fold of HDPC/Ctrl (Fig. 6A, B). With the treatment of TNF‐α for 30 min., all groups exhibited phosphorylated‐p65^S536^, but surprisingly, IκBα protein was rapidly diminished (Fig. 6C, D). Panx3 overexpression significantly decreased phosphorylated‐p65^S536^ expression and suppressed IκBα degradation compared with HDPC/Plvx (Fig. 6C). By contrast, Panx3 knockdown increased the degradation level of IκBα and increased phosphorylated‐p65^S536^ expression upon TNF‐α stimulation for 30 min. (Fig. 6D). In addition, the immunofluorescence assay showed that the nuclear translocation of p65 in HDPC/shRNA cells upon TNF‐α stimulation for 30 min. was increased compared with HDPC/Ctrl, supporting above results (Fig. 6E).

To further clarify the connection between Panx3 and NF‐κB signalling pathway, the cells were pre‐treated with BAY 11‐7082 (2 μM) for 30 min. before TNF‐α stimulation for 24 hrs, and then pro‐inflammatory cytokines were detected. The exaggerated effects of Panx3 knockdown on TNF‐α‐induced inflammatory cytokine expressions were attenuated by BAY 11‐7082 (Fig. 6F–G), indicating that Panx3 knockdown aggravated TNF‐α‐induced inflammatory response *via* activating NF‐kB signalling pathway.

## Discussion

Panx3, the third member of Panxs family, is involved in the regulation of dynamic biological functions and plays a critical role in pathogenesis of many diseases 22, such as saturated fatty acid‐induced inflammation 16, osteoarthritis 23 and dentin hypersensitivity 19. However, the effect of Panx3 in defined cell populations on pulpitis remains unascertained. Focusing on the effect of Panx3 in dental pulp inflammation, we showed that Panx3 was decreased in inflamed dental pulp tissues and Panx3 level was down‐regulated by TNF‐α, indicating that Panx3 might participate in pro‐inflammatory response of HDPCs. The overexpression of Panx3 was involved in a significant suppression of IL‐1β and IL‐6 echoed by inactivation of the NF‐κB pathway. In supporting the concept that Panx3 was directly involved in the pro‐inflammatory capacity of HDPCs, Panx3 knockdown by shRNA increased the cytokine production.

Considering the variety of cells coexisting within human dental pulp tissues (odontoblasts, immune cells, fibroblasts, neurons and so on), it is complicated to dissect the accurate role of HDPCs in the immune cells infiltration of inflamed dental pulp tissues. Therefore, only cell culture paradigms allow for the confirmation of their response to defined inflammatory stimulation. As might be expected, our experiments showed that TNF‐α depressed the expression of Panx3 in a dose‐dependent manner. TNF‐α, both trauma and bacteria could induce its secretion, act as a pro‐inflammatory cytokine and aggravate inflammatory response, then ultimately initiate dental pulp inflammation and pain 10, 11, 12, 13, 24. Previous studies have reported that TNF‐α could suppress the expression of several hemichannels in various cell types 25, 26, 27. Despite these extensive data, the mechanism by which TNF‐α suppresses Panx3 remains unclear.

NF‐κB, a transcription factor participated in immune responses, is the key factor for TNF‐α‐mediated inflammation 28, 29. With this background in mind, we first examined the regulation of Panx3 by NF‐κB pathway. Surprisingly, it was found that the pre‐treatment of HDPCs with BAY 11‐7082, a NF‐κB pathway inhibitor, failed to prevent the TNF‐α‐induced inhibition of Panx3, but rather exacerbate the inhibitory effect. These results echoed previous study that palmitate‐induced expression of Panx3 in myotubes was mediated by TLR4‐NF‐κB 16. Using Genomatix MatInspector software, we found that there are three putative NF‐κB binding sites, which is the consensus sequences composed of GGRNNYYC (where R represents purine, Y represents pyrimidine and N represents any base) on the Panx3 promoter 30. These results indicated that NF‐κB, rather than exerting inhibitory effects, was more likely to play part in a balancing way to withstand the inhibitory effect of TNF‐α on Panx3. As a result, the hypothesis that Panx3 is regulated by NF‐κB in TNF‐α‐induced inflammatory response was rejected.

Conversely, the TNF‐α‐induced down‐regulation of Panx3 in HDPCs was rescued partly by the proteasome inhibitor, MG132. It is well known that the ubiquitin–proteasome pathway is involved in regulating multiple intracellular proteolysis 31. MG132, a peptide aldehyde that efficiently inhibits the proteolytic activity of the 26S proteasome, has been used as a selective proteasome inhibitor to elucidate the actions of the ubiquitin–proteasome pathway in various cellular processes 26, 31, 32. Hu *et al*. recently reported that inducible proteolysis has also been established to be crucial in the mechanisms for TNF‐α‐regulated intracellular signalling 33, including participating in the down‐regulation of gap junction/hemichannel, Cx43, in various cell types 25, 26, 27, 34. To the best of our knowledge, Panxs and connexins (Cxs) shared a high degree of similarity of predicted structure 35, implying an imaginable overlap in degradation. During dental pulp inflammation, TNF‐α could induce the release of MMPs, which are responsible for the degradation of collagen in dental pulp tissues 36, 37. Hence, TNF‐α probably contributes to the progression of dental pulp inflammation *via* activating both extracellular and intracellular pathways of protein degradation in the dental pulp tissue.

To verify the role of Panx3 in inflammatory response, Panx3 overexpression and knockdown were constructed by lentiviral systems harbouring Panx3 cDNA and shRNA, followed by TNF‐α challenge. During multifarious inflammatory activities, a positive feedback loop leads to the secretion of inflammatory cytokines (*e.g*. IL‐6, IL‐1β) that exaggerate the inflammation response 5, 38. It has been established that the expression of IL‐1β inflamed pulp fibroblasts is 2.5‐fold higher than that in healthy pulp fibroblasts 39. Similarly, the level of IL‐6 in diseased human dental pulp tissues was dramatically increased compared with normal pulps 40. These findings demonstrated that those cytokines might be involved in pulpal inflammation 41. The levels of IL‐1β and IL‐6 in primary HDPCs stimulated by TNF‐α were enhanced after Panx3 knockdown, whereas Panx3 overexpression significantly inhibited them. Taken together, this phenomenon indicated that Panx3 could protect dental pulp cells from inflammation *via* modulating inflammatory cytokines production.

The next question that we addressed was how Panx3 regulate inflammatory response. We switched our mindset and concentrated on the effect of Panx3 on NF‐κB signalling pathway. NF‐κB family comprises of five members: p50/p105 (NF‐κB1), p52/p100 (NF‐κB2), p65 (RelA), RelB and c‐Rel. In an inactivated state, the canonical form of NF‐κB is a heterodimer, consisting of two members, p65 and p50/p105, and bound to IκB protein, an inhibitory family existing in the cytoplasm 42. When TNF‐α binds to its receptor, the IκB kinase (IKK) complex is switched on and then phosphorylates IκB protein, marking the degradation of IκB 43. Meanwhile, NF‐κB dimmers escape from IκB for translocation into the nucleus, where they activate numerous genes involved in inflammation. In addition, post‐translational modifications also cause functional alteration of NF‐κB pathway, such as the phosphorylation of NF‐κB p65 present to be activated modifications 44. In this study, Panx3 overexpression inhibited TNF‐α‐induced NF‐κB transcriptional activity, suppressed TNF‐α‐stimulated phosphorylated‐p65^S536^ expression, decreased IκBα degradation and diminished the nucleus translocation of p65. On the contrary, Panx3 knockdown played an opposite effect. Moreover, pre‐treatment of Panx3 knockdown HDPCs with BAY 11‐7082 substantially antagonized TNF‐α‐triggered cytokine expression. However, the molecular mechanism of this cellular event remains unclear. One possible explanation is that Panx3 acts as an interacting partner of BCL6 30, which is a transcriptional repressor and could suppress NF‐κB activity in several circumstances 45, 46. In addition, BCL6 plays critical roles of immunity and inflammation in various cell types 47. The interaction of Panx3 with BCL6 would be likely to explain Panx3‐mediated repression of NF‐κB. A second possibility is that overexpression of Panx3 in HDPCs result in an increase in ATP secretion, which leads to a decrease in cAMP/PKA 48. Binding of cAMP to regulatory subunits (PKAr) causes an allosteric change in kinase complex and releases free active catalytic subunits (PKAc). PKAc has been obviously believed to phosphorylate p65 at Ser276, thus activating NF‐κB 49, 50, 51. Therefore, we can speculate that overexpression of Panx3 suppresses NF‐κB signalling pathway *via* inhibiting cAMP/PKA signalling. Consequently, it is plausible that the regulation of NF‐κB by Panx3 could be contributed to the function of Panx3 (hemichannel function) or interactions with other proteins, such as BCL6. In addition, these observations above mentioned might reveal a negative feedback between Panx3 and NF‐κB transduction pathway.

**Figure 7:**
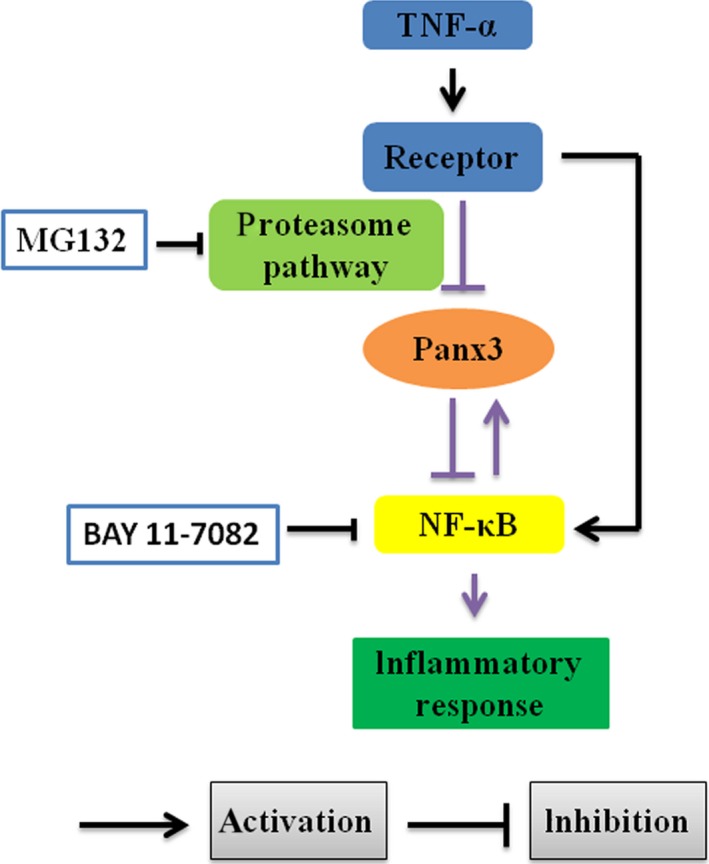
Schematic illustration of the role of Panx3 in dental pulp inflammation. TNF‐α could down‐regulate the expression of Panx3 *via* activating proteasome pathway, meanwhile the NF‐κB might balance the effect. In addition, Panx3 could suppress NF‐κB signalling pathway, leading to inhibition of TNF‐α‐induced inflammatory response. The black lines indicate that the interactions have been clearly established in previous studies. The purple lines indicate the connection established in this study. MG132, a proteasome inhibitor; BAY 11‐7082, a NF‐κB inhibitor.

In this study, we explored the intrinsic mechanisms of HDPCs participating in dental pulp inflammation. Inflammatory stimulation leads to Panx3 dysfunction *via* proteasome pathway, which may result in positive regulation of inflammatory response through excessive activation of NF‐κB. Meanwhile, NF‐κB pathway also acts in a balancing manner during Panx3 degradation (Fig. 7). Taken together, Panx3 was firstly proved to be a negative regulator of TNF‐α‐mediated inflammatory response and may be a therapeutic target of dental pulp inflammation. Nonetheless, further investigation on the direct interaction between Panx3 and NF‐κB warrants further exploration as well as whether this interaction occurred in other cells. In addition, whether the observed anti‐inflammatory effects of Panx3 was a consequence of a systematic Panx3 overexpression or addition of specific Panx3 function in HDPCs needs to be distinguished in further study.

## Conflict of interest

The authors confirm that there are no conflicts of interest.
